# From lows to highs: using low-resolution models to phase X-ray data

**DOI:** 10.1107/S0907444913022336

**Published:** 2013-10-18

**Authors:** David I. Stuart, Nicola G. A. Abrescia

**Affiliations:** a Division of Structural Biology, The Wellcome Trust Centre for Human Genetics, University of Oxford, Roosevelt Drive, Headington, Oxford OX3 7BN, England; b Diamond Light Source Ltd, Diamond House, Harwell Science and Innovation Campus, Didcot, England; c Structural Biology Unit, CIC bioGUNE, CIBERehd, Bizkaia Technology Park, Bld 800, 48160 Derio, Spain; d IKERBASQUE, Basque Foundation for Science, 48011 Bilbao, Spain

**Keywords:** virus structure, phasing methods, data collection, noncrystallographic symmetry

## Abstract

The study of virus structures has contributed to methodo­logical advances in structural biology that are generally applicable (molecular replacement and noncrystallographic symmetry are just two of the best known examples). Moreover, structural virology has been instrumental in forging the more general concept of exploiting phase information derived from multiple structural techniques. This hybridization of structural methods, primarily electron microscopy (EM) and X-ray crystallography, but also small-angle X-ray scattering (SAXS) and nuclear magnetic resonance (NMR) spectroscopy, is central to integrative structural biology. Here, the interplay of X-ray crystallography and EM is illustrated through the example of the structural determination of the marine lipid-containing bacteriophage PM2. Molecular replacement starting from an ∼13 Å cryo-EM reconstruction, followed by cycling density averaging, phase extension and solvent flattening, gave the X-ray structure of the intact virus at 7 Å resolution This in turn served as a bridge to phase, to 2.5 Å resolution, data from twinned crystals of the major coat protein (P2), ultimately yielding a quasi-atomic model of the particle, which provided significant insights into virus evolution and viral membrane biogenesis.

## Abstract 2

An unusual example of how virus structure determination pushes the limits of the molecular replacement method is presented.

## 1. Introduction

Tough structural problems, especially those relating to viruses, have from their very infancy required a combination of techniques such as electron microscopy (EM), X-ray crystallography and small-angle X-ray scattering (SAXS) (Stanley, 1935 ▶; Bawden & Pirie, 1938 ▶; Leonard *et al.*, 1953 ▶; Schmidt *et al.*, 1954 ▶; Crick & Watson, 1957 ▶; Kruger *et al.*, 2000 ▶). Recently, EM and X-ray crystallography have taken the leading role in the development of hybrid methods (Chiu & Smith, 1994 ▶; Rossmann, 2000 ▶; Gilbert *et al.*, 2003 ▶; Rossmann *et al.*, 2005 ▶; Johnson, 2008 ▶; Steven & Baumeister, 2008 ▶), although useful techniques now also include nuclear magnetic resonance (NMR) spectroscopy and mass spectrometry (MS). Together, these methods are helping to realise a vision of the cellular landscape spanning a continuum in the ångström to nanometre resolution range (Badia-Martinez *et al.*, 2013 ▶).

It has become common practice to provide quasi-atomic models by fitting the X-ray crystal structures of individual components determined at near-atomic resolution into a lower resolution density map (EM or X-ray derived) of an intact complex (Rossmann, 2000 ▶; Gilbert *et al.*, 2003 ▶). Despite this, it is interesting to note that there remain rather few examples where high-resolution X-ray data have been phased starting from low-resolution EM reconstructions (Trapani *et al.*, 2010 ▶).

**Figure 1:**
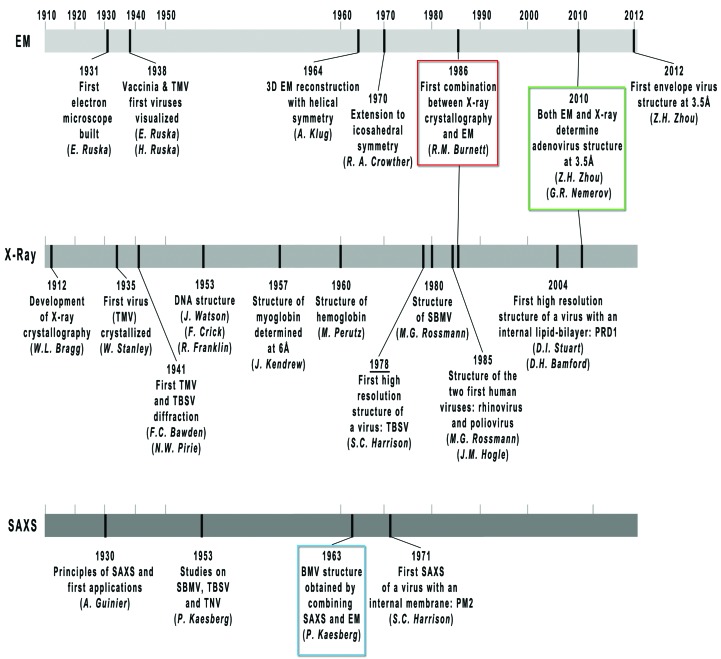
Timeline of the major achievements in structural virology by EM, SAXS and X-ray crystallographic techniques for the determination of virus structures. TMV, *Tobacco mosaic virus*; TNV, *Tobacco necrosis virus*; TBSV, *Tomato bushy stunt virus*; PRD1, lipid-containing bacteriophage PRD1; SBMV, *Southern bean mosaic virus*; BMV, *Bromegrass mosaic virus*; PM2, lipid-containing bacteriophage PM2. Coloured boxes mark those events where EM, X-ray or SAXS have been successfully combined or have provided equivalent resolution. Names beneath each landmark correspond to the author responsible for the accomplishment. Owing to their relevance, the DNA and myo- and hemoglobin structures are also included. Adapted from Badia-Martinez *et al.* (2013 ▶, where full references are given) with kind permission from Springer Science+Business Media BV.

Major contributions to these developments have come from the study of icosahedral viruses. Certain viruses are relatively easy to prepare (much early work used plant viruses, which are available in very large amounts) and crystallize, yielding crystals with high noncrystallographic symmetry (NCS), since the fivefold axes of an icosahedron cannot be accommodated in a crystal lattice. For these reasons, structural virology has played an important role in the development and consolidation of the molecular-replacement (MR) and NCS-averaging methods for phase determination (Rossmann & Blow, 1962 ▶; Harrison & Jack, 1975 ▶; Harrison *et al.*, 1978 ▶; Rossmann, 1990 ▶). Milestones are shown in Fig. 1 ▶.

Here, we briefly review the contributions of structural virology to the development of the MR technique and then describe, as an example, the MR procedures that have led to the quasi-atomic structure of the marine internal membrane-containing bacteriophage PM2 using low-resolution cryo-EM and X-ray models.

## 2. Structural virology and molecular replacement

The structure determination of the tetrameric enzyme glyceraldehyde 3-phosphate dehydrogenase (GAPDH) provided the first illustration of successful phasing by a combination of NCS and isomorphous replacement (Buehner *et al.*, 1974 ▶), although an indication of the potential can be seen in the 1964 paper on α-chymotrypsin (Blow *et al.*, 1964 ▶). The GAPDH system was also the first example of phasing *via* the use of a molecular model or envelope as a search probe (interestingly, attempts at phasing GAPDH using spherical rather than molecular envelopes failed; Rossmann & Arnold, 1993 ▶). However, the idea of exploiting the high 532 point symmetry of icosahedral viruses to solve the Patterson function dates back to a similar time (Argos *et al.*, 1975 ▶). Indeed, the first two virus structures solved by X-ray crystallography (Fig. 1 ▶), *Tomato bushy stunt virus* and *Southern bean mosaic virus*, used MR phases to locate the heavy-atom substructure and prime the low-resolution isomorphous replacement phasing (Harrison *et al.*, 1978 ▶; Abad-Zapatero *et al.*, 1980 ▶). Soon after that, the first examples of successful phase extension of initial MR phases obtained with virus structures with almost no sequence identity (Acharya *et al.*, 1989 ▶) or even spherical envelopes (Tsao *et al.*, 1992 ▶) followed. This MR approach, however, had (and still has) to take into account the eventuality of phases converging to the Babinet-inverted phase solution (180° out of phase from the correct phases), a particularly risky circumstance when starting from very low resolution model phases and when using highly symmetrical envelopes (Tsao *et al.*, 1992 ▶; Plevka *et al.*, 2011 ▶).

## 3. Phase interplay between EM, SAXS and X-ray crystallography

**Figure 2:**
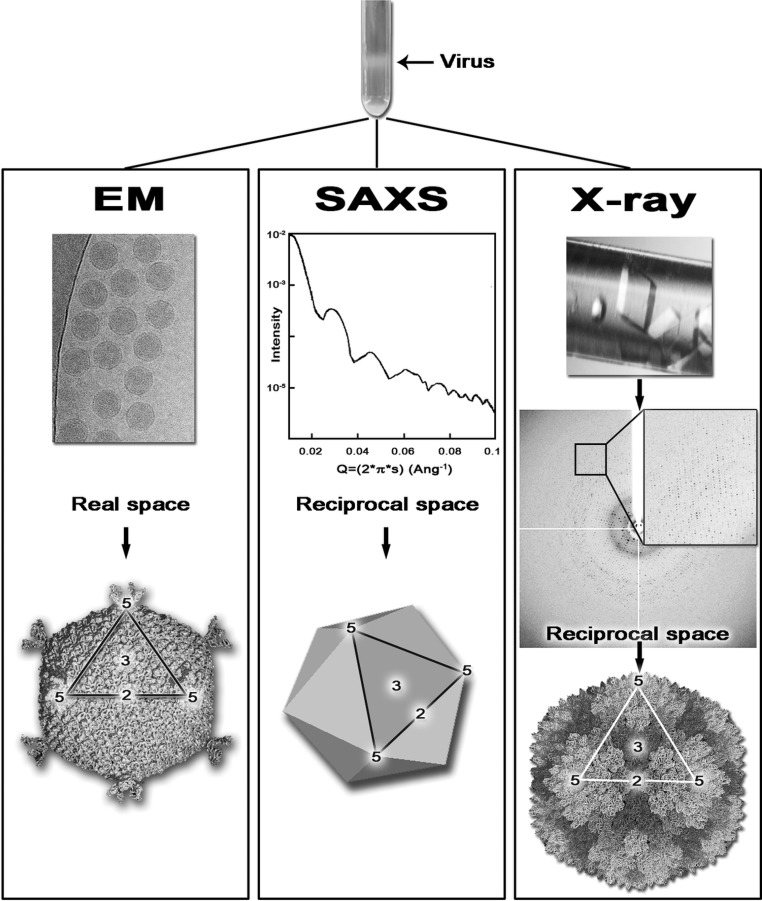
Schematic representation of the workflow of virus structure determination in EM, SAXS and X-ray crystallography. The black arrow at the top indicates the band corresponding to the virus particles following ultracentrifugation. In EM the purified virus is placed on a microscopy grid, flash-cooled and the two-dimensional projections of the virus are then visualized in an electron microscope, producing real-space images (top); post-processing of these images provide the virus structure (bottom). In SAXS the purified virus in solution is irradiated by an X-ray beam producing a low-angle scattering curve (top) containing raw data in reciprocal space; post-processing of the data produces the overall shape and molecular architecture (bottom) of the virus. In X-ray crystallography the purified virus is used to obtain virus crystals (top) that are irradiated to produce diffraction images (centre) containing raw data in reciprocal space; once the data have been processed and the phase-problem has been solved, the virus structure is obtained at atomic resolution (bottom). The outlined region in the virus models across the EM, SAXS and X-ray panels delineates one of the 20 viral facets and the numbers indicate the icosahedral symmetry axes (2, twofold; 3, threefold; 5, fivefold). Each technique provides structural information in a different resolution range, at times overlapping. Reproduced from Badia-Martinez *et al.* (2013 ▶) with kind permission from Springer Science+Business Media BV.

EM, SAXS and X-ray crystallo­graphy provide structural information at different but overlapping resolution ranges (Johnson, 2008 ▶; Steven & Baumeister, 2008 ▶; Badia-Martinez *et al.*, 2013 ▶; Fig. 2 ▶). The obvious advantage of EM and SAXS over X-ray crystallography is that they do not require a crystalline array (Fig. 2 ▶), whereas (virus) crystallography has yielded higher resolution (Fry *et al.*, 2007 ▶). However, when studying viruses, these days cryo-EM can provide three-dimensional reconstructions at ∼3.5 Å resolution (Jiang *et al.*, 2008 ▶; Liu *et al.*, 2010 ▶) for large and small viruses alike, including enveloped ones such as dengue (Zhang *et al.*, 2013 ▶; Fig. 1 ▶). This is mainly owing to the availability of increasingly powerful electron microscopes with fast, sensitive detectors and increasingly powerful computational methods (Zhou & Chiu, 2003 ▶; Bai *et al.*, 2013 ▶).

Bio-SAXS has become largely automated (Blanchet *et al.*, 2012 ▶) and whilst work on viruses has moved from their architectural characterization towards the analysis of dynamic processes (Canady *et al.*, 2001 ▶; Lee *et al.*, 2004 ▶), protein SAXS is performed mainly to elucidate molecular envelopes and the spatial arrangement of binding partners (Svergun & Koch, 2002 ▶).

Since molecular replacement works by the cross-correlation of Patterson vectors, either three-dimensional atomic models or electron-density maps can be given as search models. Thus, any structural information obtained by SAXS, EM, X-ray crystallography and NMR can, in theory, be used (for more discussion on how a one-dimensional tool such as SAXS can lead to three-dimensional results, see Svergun & Koch, 2002 ▶; Badia-Martinez *et al.*, 2013 ▶). Undeniably, the original successes in virus phasing using unrelated virus structures boosted confidence in phasing high-resolution X-ray data from low-resolution models. For instance, the structure of ornithine transcarbamoylase (OTCcase; Villeret *et al.*, 1995 ▶), using a low-resolution 8 Å X-ray model to phase and phase-extend 3 Å resolution X-ray data, was inspired by the earlier *Mengovirus* and *Foot-and-mouth disease virus* structure determination protocols (Luo *et al.*, 1987 ▶; Acharya *et al.*, 1989 ▶).

Cryo-EM and negative-stain EM low-resolution reconstructions provide, in principle, a general vehicle for the phasing of protein X-ray data, as shown in many test cases (Dodson, 2001 ▶; Navaza, 2008 ▶; Xiong, 2008 ▶). Although this approach is not routine, it should be considered whenever (i) the self-rotation function suggests the presence of multiple copies of the target protein in the asymmetric unit, (ii) low-resolution phases are available and (iii) the resolution ranges of the template and target structures overlap (Trapani *et al.*, 2010 ▶). Once the operators that relate the different protein copies have been accurately determined, the NCS is exploited to improve the starting phases and for the phase extension procedure (Rossmann, 1995 ▶; Trapani *et al.*, 2010 ▶).

Modern high-quality NMR structures can also be used as MR search probes, although they require careful preparation (Mao *et al.*, 2011 ▶). In contrast, the phasing of nitrite reductase using a molecular envelope obtained by SAXS studies was successful to only 20 Å resolution (Hao *et al.*, 1999 ▶), and to our knowledge there are no successful examples of SAXS phasing and phase-extension leading to reliable phases at high resolution. Finally, modern molecular-modelling software such as *Rosetta* can generate initial templates for MR (Terwilliger *et al.*, 2012 ▶).

## 4. A case study: the quasi-atomic structure of lipid-containing bacteriophage PM2

The marine bacteriophage PM2 (molecular mass of ∼45 MDa) is one of only two membrane-containing viruses solved by X-­ray crystallography to date (Abrescia *et al.*, 2004 ▶, 2008 ▶; Cockburn *et al.*, 2004 ▶). PM2 was crystallized by vapour diffusion in quartz capillary tubes. Data were collected from a large number of crystals directly irradiated from these capillary tubes cooled to 273 K at several synchrotron beamlines (ID14-EH1 and ID14-EH2, ESRF, Grenoble, France and PX06SA, SLS, Zurich, Switzerland; Abrescia *et al.*, 2008 ▶). In addition, virions were labelled with selenomethionine (SeMet) by finding a strain of the host (genus *Pseudoalteromonas*) which was auxotrophic for methionine, and growing in a defined rich medium. Data from these labelled particles provided important information for the final structural interpretation (Kivelä *et al.*, 2008 ▶). Finally, crystals of the isolated major capsid protein (MCP) P2 were grown using a nanoscale crystallization technique and X-ray data were collected on BM14 at ESRF, Grenoble, France (Abrescia *et al.*, 2005 ▶, 2008 ▶, 2011 ▶).

In the next two sections, we focus on those aspects of the molecular-replacement procedures used to obtain the PM2 quasi-atomic model that might provide guidance in the use of low-resolution models as templates in MR.

### 4.1. Phasing low-resolution X-ray data for the PM2 virion by molecular replacement

Data processing and scaling were carried out using the *HKL* program suite (Otwinowski & Minor, 1997 ▶) and the data were post-processed as described in Diprose (2000 ▶) and Abrescia *et al.* (2008 ▶). Analysis of the space group (*C*2) and unit-cell parameters (*a* = 946.9, *b* = 677.6, *c* = 1067.6 Å, β = 102.9°) with prior knowledge of the virion dimensions (∼600 Å; Huiskonen *et al.*, 2004 ▶) suggested the presence of one virus per asymmetric unit. The final data set was assembled from images taken from many hundreds of crystals, but as soon as we had accumulated ∼20% completeness we initiated structure determination, because in the presence of such high NCS, NCS-related spots can essentially substitute for the ones that are as yet unmeasured, and thus afford the completeness needed. We used *X-PLOR* v.3.85 (Brünger, 1992 ▶) to determine the orientation and position of the virus within the unit cell.

#### 4.1.1. Self-rotation (SR) search

**Figure 3:**
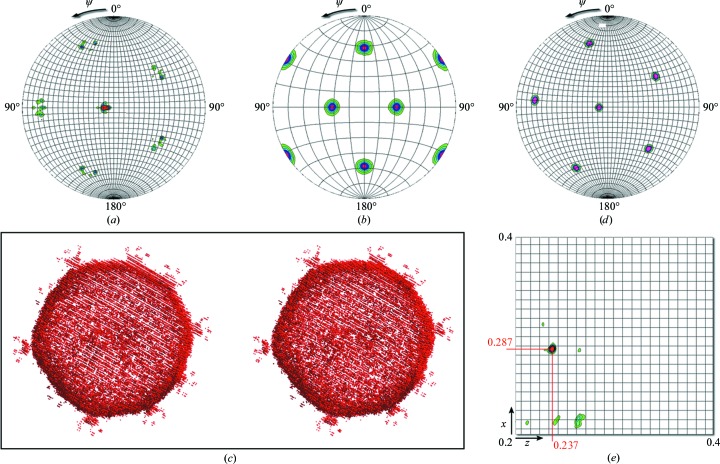
Molecular-replacement steps in PM2 structure determination. (*a*) κ = 72° section of the self-rotation function (resolution range 30–8.5 Å; integration radius 80–400 Å) showing the directions of the six fivefold icosahedral symmetry axes of PM2 within the *C*2 space-group crystals; the doubling of the peaks is owing to the fact that the virus is tilted respective to the crystallographic twofold symmetry axis and none of the twofold icosahedral symmetry axes are aligned with the crystallographic twofold. (*b*) κ = 72° section of the self-rotation function calculated using the **F**_calc_ from the PM2 pseudo-atomic model at 13 Å resolution in a *P*1 cell, confirming the initial Crowther 222 orientation of the original virus map. (*c*) Stereoview of the newly oriented pseudo-atomic model of PM2 mimicking the virus orientation within the cell. Spikes protruding from the capsid are visible. (*d*) κ = 72° section of the self-rotation function of the PM2 pseudo-atomic model in a *P*1 cell after the application of the rotation derived from (*a*) and thus replicating the orientation of the virus within the cell. (*e*) Translation function calculated between 50 and 13 Å resolution with a clear peak marking the position in fractional coordinates of the virus in the unit cell. All self-rotation and translation functions were calculated in *X-PLOR* (Brünger, 1992 ▶) and rendered using the software *GROPAT* (R. M. Esnouf, unpublished program). Contours start at 3σ and increase in 1σ intervals.

The SR function (SRF) was calculated by adapting the corresponding script (self_rf.inp) in *X-PLOR* to search for the orientation of the PM2 virion. The X-ray data resolution range used in the search spanned 30–8.5 Å. Minimum and maximum Patterson vector lengths were carefully chosen between a minimum of 80 Å and a maximum of 400 Å to include predominantly intramolecular vectors. In rotation functions the higher-order symmetry axes are seen most clearly, so we inspected the κ = 72° (in spherical polar angles) section of the SRF to determine the locations of the fivefold axes. *X-PLOR* produced a .3dmatrix file that was then rendered using the *GROPAT* software (R. M. Esnouf, unpublished program; available from the author at robert@strubi.ox.ac.uk; Fig. 3 ▶ *a*). In the hemisphere shown we would expect to see six fivefold axes for each virion in the unit cell. For space group *C*2, with a whole particle in the crystallographic asymmetric unit, two sets of peaks would be expected. Notice in Fig. 3 ▶(*a*) that since an icosahedral axis is close to the crystallographic twofold axis, it appears as if the set of six peaks is ‘split’. Notice also that despite the data being weak, low-resolution and incomplete [*I*/σ(*I*) = 4.3 overall and 1.2 in the last (8.5–8.3 Å) resolution shell], and despite having included only reflections with partiality greater than 70% (for details of the data processing, please see Abrescia *et al.*, 2008 ▶), the peaks in the SRF are sharp and well above the noise.

Next, the density from a preliminary PM2 cryo-EM reconstruction at 13 Å resolution corresponding to the capsid and spike proteins (Huiskonen *et al.*, 2004 ▶) was filled with atoms on a 3 Å grid using the *General Averaging Program* (*GAP*; D. I. Stuart & J. M. Grimes, unpublished work; software for computers running Linux is available on request from DIS). Working with this pseudo-atomic model facilitated the application of the rotations, translation and changes in scale necessary for MR in *X-PLOR.*

Firstly, this PM2 pseudo-atomic model was orientated such that the icosahedral twofold axes were aligned with the Cartesian axes (Crowther 222 setting; Crowther, 1971 ▶) and this was checked by computing the SRF using the corresponding structure factors (**F**_calc_) calculated in *X-PLOR* (model_fcalc.inp) within the same resolution range as the experimental data but in space group *P*1 (Fig. 3 ▶ *b*). Secondly, the model was rotated in such a way as to orient the fivefold axes from the initial 222 setting to the experimentally observed fivefold-axis directions (Fig. 3 ▶ *a*). The rotated model is shown in Fig. 3 ▶(*c*). To confirm the correct application of this rotation, the **F**_calc_ were calculated (in space group *P*1) and the SRF was computed (Fig. 3 ▶ *d*). As expected, this contains six peaks, each of which exactly overlaps a peak in the SRF for the experimental data in space group *C*2 (Fig. 3 ▶ *a*).

#### 4.1.2. Translation search (TS)

Having determined the orientation of the pseudo-atomic model of the virus (Fig. 3 ▶ *c*), the template virus particle in this orientation was then used to perform a translation search in *X-PLOR* using the E2E2 target function (Brünger, 1992 ▶; translation1.inp). The E2E2 correlation-coefficient target function essentially measures the fractional overlap of Patterson vectors between the experimental data and the model. Owing to the arbitrary origin along the *y* direction in the *C*2 space group, the search only needed to be performed in a single *xz* plane. Also, from packing considerations we were able to restrict the search to between 0.2 and 0.4 (fractional coordinates) in both *x* and *z*. The TS was performed with data between 50 and 13 Å resolution and produced a single unequivocal 36σ peak (correlation coefficient = 0.146; σ = 0.004) at fractional coordinates (0.286 0.000 0.237) (see Fig. 3 ▶ *e*).

To assess whether the magnification of the PM2 cryo-EM reconstruction (and consequently of the pseudo-atomic PM2 model) was in error, a check was performed by varying the scale of the pseudo-atomic PM2 model from 0.8 to 1.2 (in steps of 0.05) and re-running the translation search, monitoring the increase/decrease of the peak heights of the TS function. The values of the maximum correlation coefficient obtained during this test were 30–70% lower than the 0.146 obtained with the original scale, thus indicating no coarse magnification error of the cryo-EM map. Since these calculations were performed at low resolution, throughout the searches and the calculation of the Fast Fourier Transform (FFT) of the atomic pseudo-PM2 model, the *B*_scale_ (as an overall *B* factor added to the individual atomic *B* factors) was set to 300 Å^2^ to ‘expand’ the atoms (placed on a 3 Å grid) and to ensure that they were sampled adequately by the coarse FFT grid set to 4.6 Å (∼1/3 of the highest resolution; Brünger, 1992 ▶).

With the pseudo-atomic model safely located within the unit cell, a rigid-body refinement was carried out (*X-PLOR*; target function XREF, resolution range 50–13 Å) which refined the virus position (*R*_start_ = 58.7%, *R*_final_ = 54.6%) by the following residual rotations and translations [rotation (°) = (−0.35 −0.23 −0.37); translation (Å) = (−0.08 −0.02 −0.09)].

Phases were then determined to 13 Å resolution and the 60 NCS operators calculated and used for the phase-extension procedure. The fact that none of the icosahedral twofold axes were aligned with the crystallographic twofold axis allowed us to exploit the full 60-fold icosahedral redundancy. Real-space cycling averaging and solvent flattening were performed to 12 Å resolution, and the phases were gradually extended to 7 Å (averaging *R* factor start/final 28/23%; correlation coefficient start/final 79/88%) using *GAP* and associated software (for further details of the phase-improvement procedures, see, for example, Fry *et al.*, 1993 ▶; Grimes *et al.*, 1998 ▶; Diprose, 2000 ▶; Abrescia *et al.*, 2004 ▶, 2008 ▶). This led to a map that allowed, with the incorporation of Se positions from the SeMet-labelled virus, a tentative interpretation of the detailed structure of the viral coat proteins (Abrescia *et al.*, 2008 ▶).

### 4.2. The structure determination of P2, the MCP of the PM2 virus, using a 7.6 Å resolution electron-density map as a molecular-replacement search model

**Figure 4:**
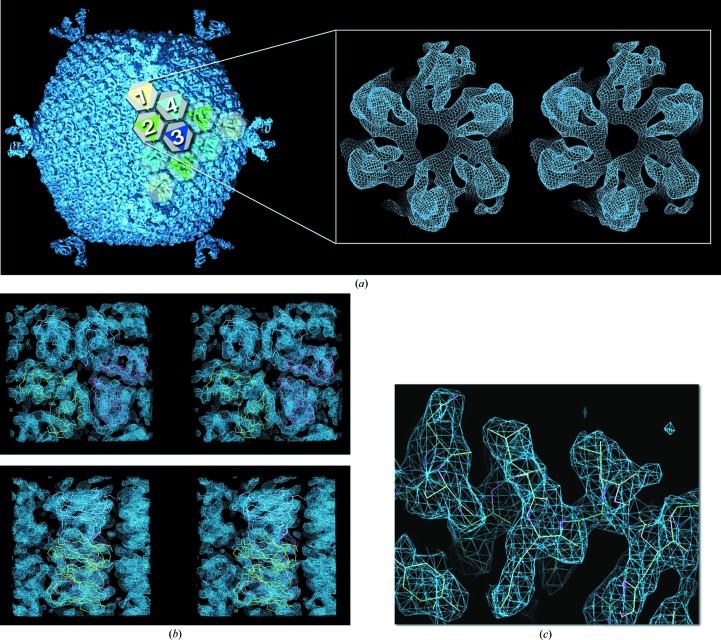

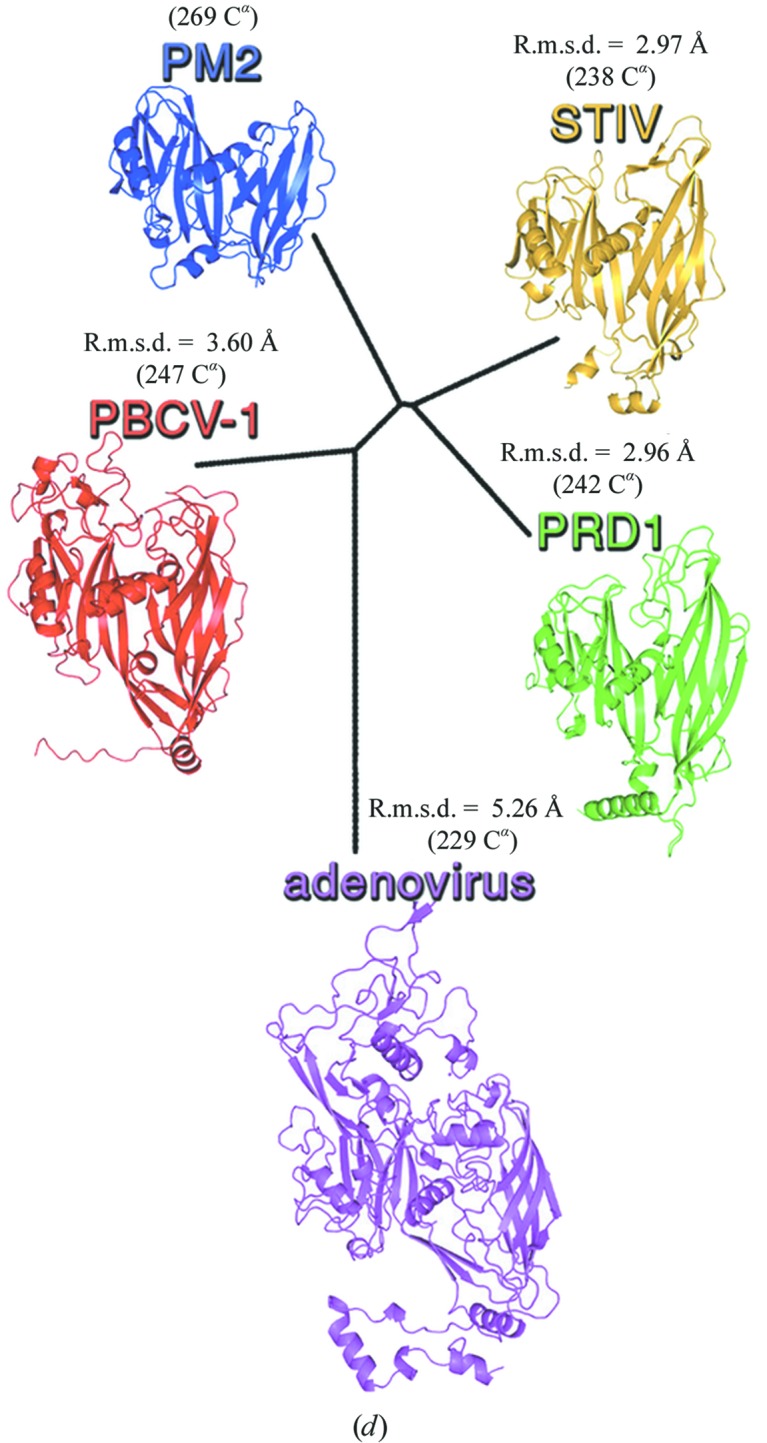
Molecular replacement in the P2 structure determination. (*a*) Averaged electron-density map (slate blue) for the entire vesicle-containing PM2 bacteriophage at 7 Å, with one triangular facet depicted with grey hexagons, indicating the pseudo-hexameric morphology displayed by the PM2 capsomers, overlaid with coloured triangles (yellow, green, blue and cyan) revealing the trimeric state of MCP P2; one icosahedral asymmetric unit is displayed as solid object and is composed of trimers 1, 2, 3 and 4 but with trimer 3 (blue) sitting on the icosahedral threefold axis, thus with a total of ten P2 subunits. Averaging the density of trimers 1, 2, 3 and 4 led to the electron density for the P2 trimer (inset as a stereoview; adapted from Abrescia *et al.*, 2011 ▶) used as a search model in *Phaser* (McCoy *et al.*, 2007 ▶). (*b*) Stereoviews of the electron density (slate blue; contoured at 1.4σ) corresponding to the MR solution obtained by *Phaser* (for clarity, only one of the two trimers composing the asymmetric unit is displayed). Top, density viewed along the threefold axis (aligned with the cell *c* axis); bottom, viewed orthogonal to the threefold axis. The C^α^ double jelly-roll model for the P2 molecules forming the trimer (cyan, yellow and red) used as an envelope for the averaging of the trimers is fitted into density. (*c*) Close-up of the experimentally phased map at 2.5 Å resolution contoured at ∼1.2σ corresponding to the α-helix spanning residues Asn63–Arg71. Reproduced from Abrescia *et al.* (2011 ▶). (*d*) Structure-based phylogenetic tree of the PM2 MCP P2 (blue) with other MCPs (available at the time) of virus members of the PRD1-adenoviral lineage (Abrescia *et al.*, 2012 ▶). The values of the C^α^ r.m.s.d. between the refined structure of P2 with each MCPs are also shown together with the C^α^ equivalences (calculated with *SHP*; Stuart *et al.*, 1979 ▶).

Despite its very high quality, the 7.0 Å resolution of the averaged map of the virus crystal structure (PDB entry 2w0c; Fig. 4 ▶ *a*; Abrescia *et al.*, 2008 ▶) could not reliably resolve the fold of the MCP P2 protein (molecular mass 30.2 kDa; 200 copies of the trimeric molecule compose the virus capsid), although the overall morphology of the capsomers suggested that the protein subunit might possess a double jelly-roll fold as observed for other viral MCPs (Benson *et al.*, 1999 ▶; Khayat *et al.*, 2005 ▶). Thus, we set out to obtain the crystal structures of the individual P2 and P1 proteins (pentamers of the latter sit at the icosahedral fivefold vertices and provide the receptor-binding site (Figs. 3 ▶ *c* and 4 ▶ *a*).

Whereas P2 was isolated and purified from the virus (Abrescia *et al.*, 2005 ▶), a range of different constructs of P1 were designed and expressed recombinantly (Abrescia *et al.*, 2008 ▶). The recombinant P1 structures were then solved by experimental phasing using SeMet-derivatized crystals (PDB entries 2vvd and 2vve; Abrescia *et al.*, 2008 ▶), whilst P2 was solved by molecular replacement in an unusual fashion (PDB entry 2vvf; Abrescia *et al.*, 2011 ▶).

Preliminary X-ray data for P2 to ∼4 Å resolution showed the presence within the crystallographic asymmetric unit of two trimers related by a translation (Abrescia *et al.*, 2005 ▶). Improved data extending to 2.5 Å resolution were obtained from an inseparable stack of several crystals. The two principal lattices were processed independently and merged, with the rejection criteria carefully set to eliminate overlapping reflections (Abrescia *et al.*, 2011 ▶). This led to a high-quality data set (the high redundancy allowed the robust detection of the overlapping reflections). Although the 7 Å resolution map of the complete virus had suggested that P2 belonged to the family of double jelly-roll MCPs, MR attempts using structurally related models such as the MCPs of PRD1 and STIV (Benson *et al.*, 1999 ▶; Khayat *et al.*, 2005 ▶) as search probes were unsuccessful (Abrescia *et al.*, 2011 ▶). Therefore, we used the 7 Å resolution electron density from the several PM2 trimers within the virus icosahedral unit as a search model. First, we averaged the electron densities of the independent P2 trimers within the icosahedral asymmetric unit (labelled 1, 2, 3 and 4 in Fig. 4 ▶ *a*; trimer 3 is actually sitting on the icosahedral threefold axis) using as a mask a double jelly-roll structure manually trimmed to roughly fill the 7 Å resolution electron-density envelope (Fig. 4 ▶ *b*). This averaged trimer map was then placed using *GAP* into a *P*1 unit cell of unit-cell parameters double the trimer diameter (*a* = *b* = *c* = 150 Å, trimer diameter of ∼74 Å; Fig. 4 ▶ *a*, inset) to ensure (i) that there would be no interatomic vectors in subsequent Patterson function manipulations and (ii) the molecular envelope was appropriately sampled (Rossmann & Arnold, 1993 ▶).

*Phaser* (McCoy *et al.*, 2005 ▶) was used for MR *via* *CCP*4 (Winn *et al.*, 2011 ▶). The program was asked to search for two trimers in the asymmetric unit with the keywords ‘EXTENT 90 90 40’ and ‘RMS 1.5’ within the resolution range 30–7 Å (the resolution was reset automatically by *Phaser* between 29.8 and 7.6 Å; the ‘EXTENT’ keyword defines the limits in *x*, *y* and *z* of the region of density to be considered; for details of *Phaser* keywords, see http://www.phaser.cimr.cam.ac.uk/index.php/Molecular_Replacement). The top peak in the fast rotation function displayed a log-likelihood gain (LLG) of 56.5 and a *Z*-score of 9.0 (the number of standard deviations above the mean). The fast translation found two sites, with the top one having an LLG of 99.0 and a *Z*-score of 8.5 (we requested peaks over 75% of the top peak). The second trimer was then searched and located. The final refinement of the top solution for both trimers (RFZ = 9.0, TFZ = 8.5, PAK = 0, LLG = 109; RFZ = 6.6, TFZ = 21.2, PAK = 0, LLG = 448; where RFZ is the rotation-function *Z*-score, TFZ is the translation-function *Z*-score and PAK is the number of packing clashes) had a negligible effect on the LLG.

Proof of the correctness of the MR solution (Fig. 4 ▶ *b*) was obtained by using phases from the *Phaser* model to calculate an electron-density map which was then used as a starting point for a phase-improvement protocol. This consisted of NCS-operator refinement prior to cyclic averaging, solvent flattening and gradual phase extension in resolution steps of 1/2000 Å using *GAP* (this step was chosen to be ∼25 times smaller than the inverse of the shortest unit-cell parameter to guarantee that no random phases would be introduced; for a mathematical formalism on the phase-extension procedure, see Rayment, 1983 ▶; Rossmann, 1990 ▶; Fry *et al.*, 1993 ▶). This process, detailed in Abrescia *et al.* (2011 ▶), led to an excellent map at 2.5 Å resolution (Fig. 4 ▶ *c*), which rendered structure determination of the double jelly-roll fold of the protein facile.

Structural superimpositions of the refined P2 atomic model with the other MCPs belonging to the same PRD1-adenoviral lineage (Abrescia *et al.*, 2012 ▶) revealed why MR using these search models failed: they showed root-mean-square deviations (r.m.s.d.) above 2.9 Å (Fig. 4 ▶ *d*), which are higher than expected for template models that are likely to succeed in MR (Terwilliger *et al.*, 2012 ▶), despite the fact that all share the double jelly-roll fold.

## 5. Conclusions

Owing to their isometric shape, icosahedral viruses (harbouring 60 symmetry-related identical building blocks) have proved to be useful for developing the MR technique. Early successes of this method using low-resolution models as search probes encouraged the adoption of a similar workflow in cases of crystallized multimeric proteins (especially where the presence of proper NCS facilitates the derivation of accurate NCS operators) and for which low-resolution structural information is available. These low-resolution phases, either derived from (cryo-)EM or X-ray crystallographic electron density, can be used as a source of initial phases for high-resolution X-ray data, easing the solution of the phase problem.

As examples of this phasing strategy, we have detailed the procedures used in the MR structure determination of the entire lipid-containing bacteriophage PM2 at 7 Å resolution and the subsequent use of this electron density in the determination of the structure of its major capsid protein P2 at 2.5 Å resolution. The resulting quasi-atomic model of PM2 illustrates the power of combining these phasing methods.
